# An Adaptive Real-Time Malicious Node Detection Framework Using Machine Learning in Vehicular Ad-Hoc Networks (VANETs)

**DOI:** 10.3390/s23052594

**Published:** 2023-02-26

**Authors:** Kanwal Rashid, Yousaf Saeed, Abid Ali, Faisal Jamil, Reem Alkanhel, Ammar Muthanna

**Affiliations:** 1 Department of IT, The University of Haripur, Haripur 22620, Pakistan; 2 Department of Computer Science, University of Engineering and Technology, Taxila 54000, Pakistan; 3 Department of Computer Science, GANK(S) DC KTS Haripur, Haripur 22620, Pakistan; 4 Department of ICT and Natural Sciences, Faculty of Information Technology and Electrical Engineering, Norwegian University of Science and Technology (NTNU), Larsgårdsvegen 2, 6009 Trondheim, Norway; 5 Department of Information Technology, College of Computer and Information Sciences, Princess Nourah bint Abdulrahman University, P.O. Box 84428, Riyadh 11671, Saudi Arabia; 6 Department of Telecommunication Networks and Data Transmission, The Bonch-Bruevich Saint-Petersburg State University of Telecommunications, 193232 Saint Petersburg, Russia; 7 Department of Applied Probability and Informatics, Peoples’ Friendship University of Russia (RUDN University), 117198 Moscow, Russia

**Keywords:** real-time malicious nodes, VANET, machine learning, DDoS, OMNET++

## Abstract

Modern vehicle communication development is a continuous process in which cutting-edge security systems are required. Security is a main problem in the Vehicular Ad Hoc Network (VANET). Malicious node detection is one of the critical issues found in the VANET environment, with the ability to communicate and enhance the mechanism to enlarge the field. The vehicles are attacked by malicious nodes, especially DDoS attack detection. Several solutions are presented to overcome the issue, but none are solved in a real-time scenario using machine learning. During DDoS attacks, multiple vehicles are used in the attack as a flood on the targeted vehicle, so communication packets are not received, and replies to requests do not correspond in this regard. In this research, we selected the problem of malicious node detection and proposed a real-time malicious node detection system using machine learning. We proposed a distributed multi-layer classifier and evaluated the results using OMNET++ and SUMO with machine learning classification using GBT, LR, MLPC, RF, and SVM models. The group of normal vehicles and attacking vehicles dataset is considered to apply the proposed model. The simulation results effectively enhance the attack classification with an accuracy of 99%. Under LR and SVM, the system achieved 94 and 97%, respectively. The RF and GBT achieved better performance with 98% and 97% accuracy values, respectively. Since we have adopted Amazon Web Services, the network’s performance has improved because training and testing time do not increase when we include more nodes in the network.

## 1. Introduction

The Vehicular Communication System (VCS) increased with time, especially when Tesla self-driving cars entered the VANET market. The computational power and communication between these vehicles increase with time, and malicious attacks are also increased to harm the data generated through these devices [1]. According to the research, almost one-third of the people in the developed world have a vehicle license to drive cars. The vehicles in VANET use wireless technology, such as 5G, that enables attackers to attack the network using its open nature. As a prevention to this attack, misbehavior detection systems are developed to attack and prevent these behaviors. The prevention only captures the incoming data packets and provides an easy way to enable these attackers to catch and enlist them for distribution. Many attacks on the VANET environment can hack the network [2]. In this chapter, we introduce our research with some used infrastructure and the ability to communicate and handle the communication infrastructure—we discussed the motivation, problem statement, research questions, and research outline.

The VANET term is implemented from the autonomous network transformation to implement the nature of the wireless network. It holds the new generation of technologies that embrace the nature of network deployment and provides the ability to enhance knowledge and experience of network boundaries. The idea of the VANET is that it connects in the same way as the computing connectivity of mobile devices. These devices are connected to deploy the proper network architecture. Intelligent Transportation System (IST) is one of the key deployment areas that makes the network architecture more secure and provides reliable features and delivery services for the network’s needs. In VANET, we contain three types of communications, i.e., vehicle-to-vehicle (V2V), vehicle-to-infrastructure (V2I), and infrastructure-to-infrastructure (I2I) communication [3].

ITS uses the ICT concepts with data transmission flow with abilities to enhance the knowledge and the work experience of these professional skills, and the demand for nature perspectives. The ITS technology is efficient, smarter, and suitable for remote communication in VANET [4]. The Roadside Units (RSUs) and Base Stations (BS) are the common ways to provide effective delivery services of the common protocols and enhance the recovery and efficiency of policies inside the network architecture. The modern communication infrastructure makes the role more important, as others cannot do this from a development perspective. The safety of the drivers and passengers in the VANET is a high priority in this autonomous industry [5]. Several possible attacks on the VANET make its distribution more questionable regarding data transparency and security. The data is very important in the VANET. Denial of services, jamming, malware, Blackhole, spamming, Sybil, tunneling, GPS spoofing, and traffic analysis attacks are the common attacks on the VANET environment. The most significant and dangerous attack is a denial of service or distributed denial of service attack (DDoS) in VANET. These attacks make the network more vulnerable and provide a separate attack scenario for the development perspectives of the network and autonomous industry. The attacks encountered under these umbrellas destroy the network activities [6]. VANET has multiple categories, such as cloud ad hoc networks (CANET), mobile ad hoc networks (MANET), and vehicular ad hoc networks (VANET). All of these can be compromised using the attack scenario presented in this context for the VANET to be updated according to the wireless technologies. Hop-to-hop communication is allowed, or vehicle-to-vehicle communication, in this research that corresponds to secure communications using the machine learning approach. The VANET offers multiple ways to control and provides the significance of handling these types of network attacks. Multiple wireless channels are imported into the VANET that demonstrate the network architecture, make the network more secure and not vulnerable to attacks, and provide the latest coverage area to design and develop for wireless communication technologies [7].

Real-time malicious nodes are a critical concern in vehicular ad hoc networks (VANET) because they can cause severe disruption and pose a significant threat to the security and privacy of communication. VANETs rely on communication between vehicles and infrastructure to ensure smooth and safe transportation. Malicious nodes can exploit these communication channels to launch attacks on the network and cause widespread damage [8]. The importance of real-time malicious nodes in VANETs lies in their ability to compromise the network’s security and privacy. Malicious nodes can exploit network vulnerabilities and steal sensitive information, such as vehicle location, speed, and driving habits. They can also send false messages, which can cause confusion, accidents, and even gridlock on the roads.

Moreover, real-time malicious nodes can launch denial of service (DoS) attacks, which can significantly disrupt the network’s operation [9]. DoS attacks can cause communication breakdowns, leading to delays in emergency services, traffic congestion, and accidents. Therefore, it is essential to detect and isolate real-time malicious nodes in VANETs to ensure the network’s security and reliability. VANETs need advanced security protocols to detect and prevent attacks from malicious nodes. Security measures such as encryption, authentication, and intrusion detection systems are necessary to mitigate the impact of real-time malicious nodes in VANETs [10].

**Figure 1:**
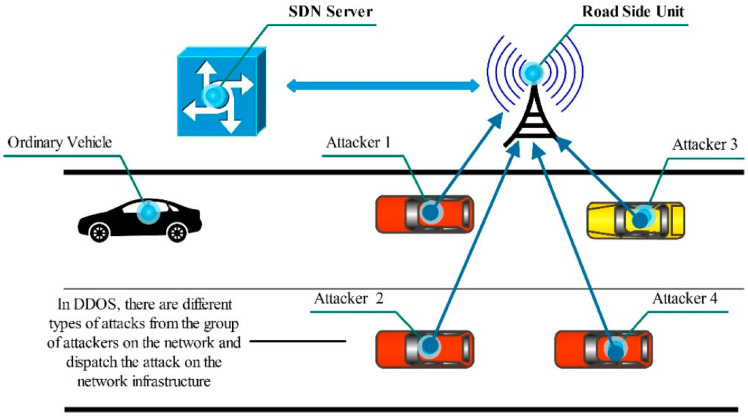
DDoS Attack on vehicles in the VANET environment [12].

In DDoS, intruders use multiple vehicles to target the network from different locations, disturbing the network density and integrity. However, all of these vehicles involved in DDoS attacks are unaware they are utilizing the attack on the network devices. All of these vehicles are also called “zombies” in the DDoS context. The results of DDoS attacks can be sewers, such as loss of human lives, accidents to automatic vehicles, and infrastructure loss. As multiple zombies are involved in this attack, detection is difficult, and there is no proper conveyancing [11]. Figure 1 shows the attack on vehicles using DDoS. In this DDoS attack from Figure 1, the attackers use other vehicles to attack single data or a major vehicle under this attack. The targeted vehicles stop working and leave for another environment. The victim vehicle did not correspond to these changes and started to drop out of the responding phase in the network. In these theses, we focused on the real-time detection and mitigation of DDoS attacks using machine learning.

**Figure 2:**
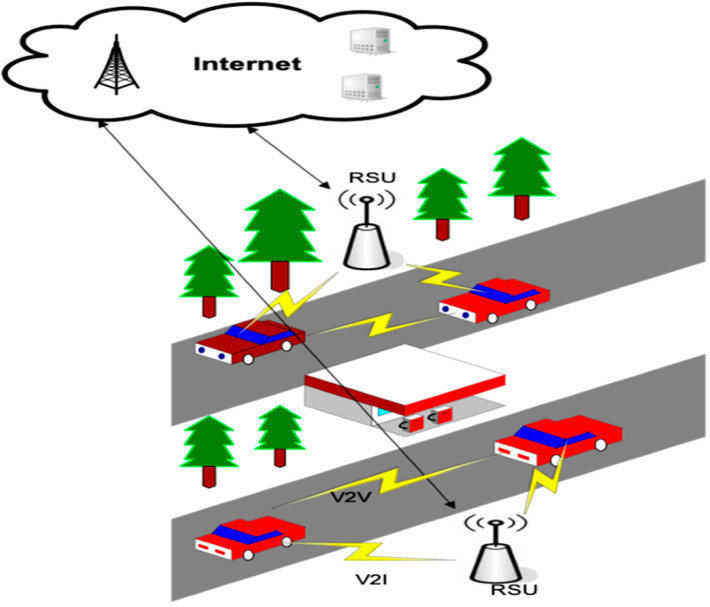
Communication architecture under the VANET environment [16].

VANET is one of the wireless multi-hop network (WMN) cases. This network provides fast technology change in different infrastructure management and enhances the work of different resources under a high level of mobility [13]. In VANET, the devices are equipped with wireless communication devices and computing technologies for inter-vehicle and intra-vehicle communication. Inter-vehicle communication, in the form of caching, is one of the promising fields of research in these development perspectives for standardization, research, and development technologies [14]. “VANETs enable a wide range of applications, such as prevention of collisions, safety, blind crossing, dynamic route scheduling, real-time traffic condition monitoring, etc. Another important application for VANETs is providing Internet connectivity to vehicular nodes. Figure 2 shows an example of a VANET”. From Figure 2, we adopted the communication using 802.11 for the V2V, V2I, and I2I communication architecture. The vehicles, RSU, and based stations use the communication architecture to explain the current scenario [15].

**Figure 3:**
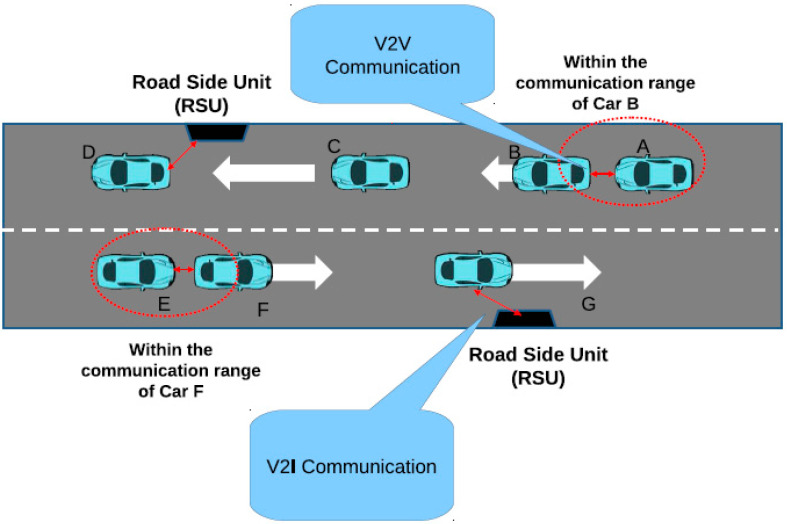
Communication in VANET using V2V, V2I, and I2I [19].

VANET provides three main components, which include the application unit (AU), on-board unit (OBU), and roadside unit (RSU) [17]. Figure 3 provides the effective communication range for VANET architecture. The architecture shows that during the V2V communication, two vehicles communicate with each other, i.e., vehicles A and B. During V2I communication, the architecture is communicated with the vehicle, i.e., RSU is communicating with vehicle G. The communication range is defined under every vehicle, based on the area surrounding the vehicle, in which it communicates with other vehicles or infrastructure [18].

Machine learning has recently been a hot topic, and its application in vehicular ad hoc networks (VANETs) has gained much attention. VANETs are an emerging technology that enables vehicles to communicate with each other and the road infrastructure to improve road safety and traffic efficiency. However, the success of VANETs depends on the reliability and efficiency of communication between vehicles, which can be affected by various factors such as signal interference, dynamic network topology, and unpredictable mobility patterns [20]. Machine learning techniques can be applied to VANETs to overcome these challenges and enhance the network’s performance. One of the main applications of machine learning in VANETs is predicting vehicles’ behavior on the road. Machine learning algorithms can analyze the data collected from various sensors and communication devices installed in the vehicle to learn driving behavior patterns, such as speed, acceleration, and lane changes. This information can then be used to predict the future behavior of the vehicle and improve communication between vehicles [21]. The machine learning algorithm is very effective at using straight learning in the machine to provide effective and quality-based training. It supports algorithms, deep learning, and AI, and shows classification and detection accuracies in the network. We adopted ML in our model to train the network to detect DDoS-compromised vehicles [22].

### 1.1. Real-Time Malicious Node Detection

VANET is always vulnerable to attacks due to its open wireless access. The wireless network is susceptible to attacks [23]. VANET is also vulnerable due to wireless technology. In VANET, there is real-time communication among the vehicles, so the detection of the malicious nodes may also come in real time; otherwise, the network is always targeted with high vulnerabilities. The security requirements must be best fitted to communicate and provide real-time malicious node detection using the scenario [24]. Malicious node detection comes with confidentiality, non-repudiation, integrity, authentication, and privacy to protect against attackers and intruders. In our case, we encounter real-time malicious node detection using the machine learning approach. This happened due to the protection and detection of different schemes that can demonstrate and provide adequate knowledge and discovery. The main idea in this context is real-time malicious node detection. Real-time means whenever a node starts behaving differently, we encounter the machine learning algorithm that starts detection of the malicious nodes [25].

### 1.2. Importance of the Research

Malicious nodes create issues such as content alteration, non-trustable content delivery, and information flow. Malicious nodes are detected through machine learning, which enables data management operations in VANET. DDoS is one of the most critical and advance level threats for VANET. In this research, we plan to handle DDoS attacks in a real-time scenario. More specifically, when we talk about real-time traffic and content sharing in VANET, the content shared data is difficult to handle whether the real-time data is secure or not. We took the idea from machine learning, and VANET combined the handling of different incoming network traffic for the DDoS attacks. The VANET and machine learning collectively secure real-time content sharing among all the vehicles.

Although many research works have been implemented and provide secure content delivery in VANET, the real-time detection of the malicious nodes in VANET is still the main idea to be solved. This proposed work solves the malicious node’s detection issues with real-time monitoring through VANET emergency packet delivery. Malicious nodes are detected using real-time monitoring schemes through VANET. A reliable mathematical model is evolved through a machine learning malicious node detection policy. VANET is considered one of the most demanding smart environments in the recent technological era. VANET faces multiple issues such as content security and privacy, and smart V2V environment. In this research, we considered malicious node detection during the flow of information in VANETs.

This research evaluates the below objectives:

- Detection of malicious nodes through machine learning.
- Enhance the throughput of the network through the detection of malicious nodes.
- True Positive Rate (TPR), False Positive Rate (FPR), True Negative Rate (TNR), and False Negative Rate (FNR) classification for malicious node detection.
- This research will contribute to secure the lives of patients when medical data is secured.
- This research enhances the security of special coveys and government data.

### 1.3. Problem Description

VANET is considered one of the most demanding smart environments in the recent vehicular technological era. VANET faces multiple security issues, such as malicious nodes, behavior changes, security, content privacy, and message exchange. Through the literature study, we learned that malicious node detection is a very state-of-the-art research gap in VANET; after studying the topic, we have seen that multiple techniques are presented on this topic, but based on our further study, we concluded that real-time malicious node detection using machine learning is one of the most critical research problems. A novel technique is presented in this research to detect and classify malicious nodes using machine learning techniques. In this research, we have considered malicious node detection during the information flow in VANET. To address the issue, we proposed a real-time machine learning-based secure information flow mechanism to enhance the privacy and security in VANETs.

In this paper, we have considered the below-mentioned questions during the task scheduling:

(i). How are malicious nodes detected in VANET in real-time?
(ii). How to achieve privacy and security of VANET under network attack?
(iii). How to deliver real-time content to vehicles in VANET using machine learning techniques?
(iv). How to deal with malicious requests generated from malicious nodes?

### 1.4. Research Contributions

The main contributions of the research are to detect malicious attacks using machine learning techniques.

(i). The research supports the real-time communication between vehicles in VANET to detect malicious node detection.
(ii). The proposed multi-layer classifier technique, called the DDoS detection scheme, detects malicious nodes that hijack a vehicle in the network using a monitoring approach.
(iii). The machine learning models are evaluated to adopt real-time malicious node detection with OMNET++ and SUMO simulations under a higher accuracy of 99%.
(iv). We design a cluster-based VANET architecture in which the RSU handles and implements the real-time machine Learning algorithms to detect the malicious nodes. This technique is conducive to overcoming the malicious activities of DDoS attacks in VANET.
(v). The proposed technique predicts real-time DDoS malicious activities by continuously monitoring each vehicle in the network using an ML algorithm.
(vi). The real-time dataset is monitored to evaluate each node through the RSU-based technique for the performance evaluation and enhancement of parameters.
(vii). The ML-based model is trained using real-time traffic analytics in VANET after observing each node’s behavior, message threshold, speed, packet routing, and network congestion and handling parameters. After the evaluation and dataset of these parameters, the ML-based algorithm is trained and tested in a real-time environment through a simulation setup.
(viii). The anomalies in the network are checked, and malicious vehicles are removed from the network using a RSU-based real-time checking mechanism.
(ix). We design a real-time malicious node detection mechanism to detect the DDoS attack nodes from real-time analysis to perform the selected performance based on a generated dataset under the severity of 10% to 70% under attack.
(x). The performance is compared with the existing state-of-the-art techniques using machine learning on a training and testing dataset model that is real-time generated under the supervision of RSU that implements the machine learning models. The network throughput and model prediction accuracy are achieved using the mentioned requirements.
(xi). The machine learning approach for DDoS-based malicious node detection and classification.
(xii). The network throughput is achieved using machine learning techniques.
(xiii). False Positive Rate (FPR), True Positive Rate (TPR), False Negative Rate (FNR), and True Negative Rate (TNR) for malicious node detection and classification.
(xiv). The research contributes towards the modern ITS-equipped vehicles from the vehicular industry for classification and model prediction accuracy and secured medical data.

Section 2 describes the thesis background with respect to related work. We define misbehavior detection in VANET. Close related work is described in this chapter of the thesis. All the related work is effectively described and utilized in these strategies. At the start of this chapter, we define the structure, and then machine learning-based malicious node detection is determined, which provides the fully reference-based allocation and development schemes. Section 3 contains the proposed method to detect real-time malicious nodes using machine learning and provide significant results using such techniques. The neural networks are introduced with the ability to handle and provide the significance needed to provide effective resources. The three state-of-the-art algorithms are defined in this chapter. The proposed model with methodology diagram is implemented in this context to define the clear features of the proposed technique. The workflow model for the proposed methodology is also explained to detect malicious nodes using these techniques. In Section 4, we simulate our research ideas using simulation tools. We adopted machine leaning and VANET model selection to deliver these services successfully. The results are gathered, and then a discussion is performed using such techniques. The analysis and ratios are performed using these techniques. In Section 5, we provide a summary of the thesis to conclude and understand the thesis in a single heading. In Section 5, we concluded the work with future directions provided in the same context.

## 2. Literature Review

The nature of the vehicular networks is open, which means it uses wireless technology to communicate under V2V, V2I, and I2I communications. The openness makes it vulnerable to cyber-attacks, such as DDoS attacks. Regarding the wireless network’s security, some solutions are provided in this context—the literature review defined the machine learning-based DDoS attack detection using real-time scenarios. According to the survey, we studied around 50 articles on machine learning-based malicious node detection using the real-time scenario. In this chapter, we focused on the real-time scenario and found the research gap which we focused on in this research. The existing techniques for malicious node detection in VANET are studied [26].

Understanding VANET security architecture is important at the initial stages of the literature before moving on to the final decision-making. In [27], the authors write a review article to discuss VANET security. The authors review almost 114 articles on VANET security. The VANET is a soft target for attacks, such as DDoS, eavesdropping, bogus information, impersonation, and hardware tempering. The DDoS attacks are considered to target resolving issues using machine learning. In [28], the authors define the security mechanisms, such as RSA, public key encryption, and elliptic curve cryptography. These security mechanisms play an important role in the development of effective mechanism handling. In this paper, the authors continuously highlight DDoS detection in VANET. The authors in [29] propose a new security protocol that safeguards against DDoS attacks in VANET. The main steps of the proposed solution are as follows:

- DDoS detection;
- DDoS reporting;
- DDoS investigation.

The DDoS handles use the activities to detect the DDoS from the literature ordinance values. DDoS is also a form of misbehavior detection. Initially, the system detects the misbehavior, and then this detection is reported to the authority named misbehavior authority. The authority then completes the detection and provides notification about the DDoS attacks. In another research article, the developers develop a new system to develop the new misbehavior system. Another research in this regard is published in [30], in which a misbehavior reporting system is designed to develop a new system. The messages are contained from vehicles that show or misbehave in the network. The evidence containers contain vehicular information from its historical behavior. This affects the simplicity of the network and promotes network operations. The detection inside the system contains the detection containers able to manage the network operations. This container contains information regarding the errors that are reported in this context.

The dataset generation and misbehavior detection from the network is a challenging task—authors in [31] design VeReMi (Vehicular Reference Misbehavior Dataset). The authors highlight the issue related to misbehavior detection and other dataset generation systems. Different datasets are generated, and studies define the misbehavior network inside these challenges. VeReMi uses log files to detect misbehavior whenever the network starts behaving as if it is engaged in malevolent activities. In this network, the source code is for about 255 simulation rounds. It detects attacks such as network traffic and attacker densities for network analysis. In [32], the author makes claims about these kinds of datasets. According to researchers, the authors achieved great achievements, but the dataset is imperfect. Through this technique, the users cannot detect multiple attacks—the author’s continuous development of network architecture using the VeReMi dataset. The dataset has less impact than other dataset techniques.

Another technique to simulate the VANET, SUMO (Simulation of Urban Mobility), is proposed. SUMO is used in multiple research activities. It is an open-source simulation for VANET traffic simulation. The authors in [33] use the study of SUMO in VANET. The tool works in combination with NS-2/3. In [29], the authors proposed a network simulation for VANET malicious node detection under NS-2 and SUMO. SUMO imports network models for related matters. The recent SUMO projects are VABENE, iTETRIS, and CityMobil. Researchers in [30] created the GUI-based road network with publicly available tools for the simulation in VANET.

Inside the VANET, another tool that is introduced is OMNET++. OMNET++ is used for GUI-based communication. It uses C++-based architecture and provides the effective delivery of network services. The simulation and development tool helps to integrate the network connections. Authors in [34] use OMNET++ to detect DDoS attacks. Four simulated vehicles are used as a study for the research topic in this network. In DDoS, more than one attacker attacks the system. The authors use multiple attack vehicles as a zombie. Another simulation by Kaur et al. [35] uses RaeSE and OMNET++ under the attack development using the web servers. Simulated DDoS attacks were generated using random access to the network effects. In this technique, multiple routers and factors are involved in addressing the issue raised in this technique. The network access ratio and packet drop ratio are observed in this technique. There are several design parameters considered for proper environmental protection. After the malicious network node attacks, the Wireshark analysis tool is used to analyze the network. The results are quite remarkable in terms of providing effective network services.

Malicious node attackers are the threat to the VANET that provides the ability to communicate and handle the network environment. The malicious node attacks incorporated in the network, when intercepted with normal vehicles, are not encounters. The attacker encounters that the packets are captured, doped, and modified in the network. According to research in [36], the attackers attack the network using OMNET++ and SUMO. VEINS is also part of the attack environment. The maps are defined using VEINS, and other tools incorporate the network. The total of different cars and other network environments are incorporated with the network disability and efficiency. According to the study, fleet-based and distributed attacks are encountered in the study environment.

The DDoS attacks come from flooding vehicles or RSUs. The authors in [37] proposed the graphical aware flooding technique in VANET. It is similar to a technique for normal flooding attacks. The packet drop ratio is increased in this type of flooding attack in the network. The vehicle behind the vehicular communication operates in this favor of the flooding attacks for the order of the flooding. Another method named OLSR (Optimized Link State Routing) is proposed in which vehicles propose a new technique for the similarity and other main features related to the network architecture. Flooding is one of the critical methods for obtaining advanced-level communication between vehicles. Many machine learning methods are used in this subject to detect and provide DDoS, Sybil, and alert message detection techniques. The attacker wants to obtain the communication pattern to provide an effective communication environment.

The authors in [38] use the case to detect the malicious nodes using machine learning models such as logistic regression, KNN, decision tree classification, random forest, and bagging. The authors check that the decision techniques used effective learning and mitigation techniques for the final approval of the detection of misbehavior detection. The accuracy of the network is achieved and provides effective qualitative research patterns for the selection and provision of the results. The authors in [39] proposed a hybrid, trustable, deep learning model to detect malicious nodes in VANET. Communication in VANET is expensive when nodes are detected and provide efficient and effective qualitative-based trust management approaches. The authors proposed a hybrid technique using deep learning to overcome challenges such as packet loss, packet damage, routing difficulties, and software and hardware failure issues. The attacks are classified inside the VANET environment. A hybrid algorithm is used for the CH selection and hybrid optimization approaches. The optimization algorithm is used to provide the effective classification and methodology for the proper certification. In the end, 94% accuracy is achieved in detecting the malicious nodes from the VANET environment.

In [40], the authors target traffic exchange and communication in VANET. DDoS attacks are considered in the network. An intrusion detection system is one possible way to handle these attacks, but the growing needs of larger networks challenge the environment. The author uses the random forest algorithm for the posterior detection of malicious nodes using high accuracy. The detection accuracy is enhanced by providing these effective networks and other related matters to detect and classify DDoS Attacks. The proposed model enhances the machine learning results with detection accuracy and value predictions.

VANET is always available for attackers’ malicious activities due to its open nature and provides many different techniques that detect and classify malicious nodes. The authors use multiple machine learning models to detect the attacks from the system. Different machine learning algorithms detect every attack under binary classification. The accuracy shows that the attack detection technique effectively handles malicious node detection. The main limitation of the work is that this does not handle the real-time detection of malicious nodes. This means that the detection accuracy is compromised when a real-time scenario is involved [41]. Another technique proposed by authors in [42] is to detect malicious nodes as position falsification attacks. The authors consider cooperative intelligent transportation systems (C-ITS) to connect advanced technology with useful features and enhanced required terminologies. The attack is dangerous for the safety of the passengers and other related situations. It plays a vital role in detecting and mitigating the vital attack system in VANET. The authors consider three features: sender position, existence, and performance. KNN and RF models are used in this system to detect and mitigate the research-based parameters related to the advanced level significance in this regard. The results effectively show better results, but the limitation is the real-time detection of the malicious nodes under different machine learning models.

Malicious node detection in VANET is challenging, especially when working under a wireless network. Additionally, network situations such as real-time detection make it more difficult to detect malicious activities from the network. The misbehavior detection/DDoS detection technique is considered in this research. The technique observed that very little work is performed under the VANET environment. The attacked scenarios are considered on multiple intensities. Table 1 shows the summary of existing techniques with working parameters compared with existing ones.

## 3. Materials and Methods

Previous research in Section 2 has developed the research method for distributed denial of service attacks through machine learning. Our research is novel because it takes into account the real-time computational cost of the proposed model, which has not been discussed in previous research. To execute the misbehavior system, the distributed denial of distribution system encompasses the distributed system technological features, which reduces the cost of the detection of misbehavior of individual specific vehicles. Apache Spark is a technology that resolves this issue using open-source distributed technology. Common computational languages are designed to distribute useful technology features, such as R, Python, etc. The parallel operations on each cluster are primarily focused on the tool. A cluster is a group of nearby vehicular nodes with similar characteristics. After the composition of clusters, the data is transferred to a neural network to check the DDoS attack in the case of this light scenario.

### 3.1. Computation with Distributed System Implementation

**Figure 4:**
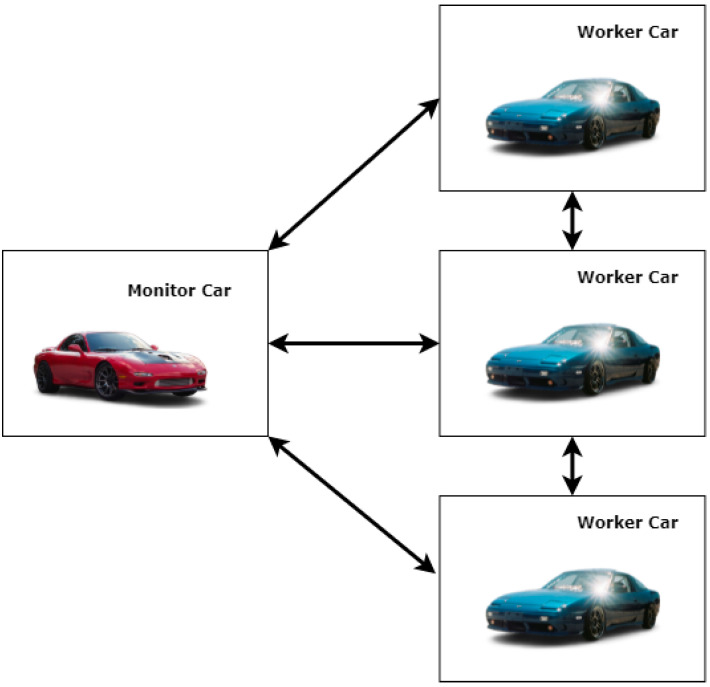
High-level implementation of the proposed vehicular distributed system implementation.

The distributed system remains a single point of the working group, which consists of multiple machines working together. In the vehicle system, we consider each machine as a vehicular system with the implementation of the complete distributed system. Each vehicle is a part of the distributed system. Every vehicle in the distributed system communicates with each other to design and use the neural network (NN) model. We can call it a distributed neural network (DNN) model. The system effectively reduces the allocation of resources on each vehicle and speeds up the detection of malicious nodes. A high-level system implementation of this proposed system is implemented in Figure 4. One vehicle is selected as a monitor vehicle to monitor the safety of a specific location. The monitor vehicle contacts other vehicles to collect malicious information or misbehavior detection. The worker vehicle is only used as a computational resource vehicle. Every new role in the network is circulated to neighboring nodes to add additional security.

**Figure 5:**
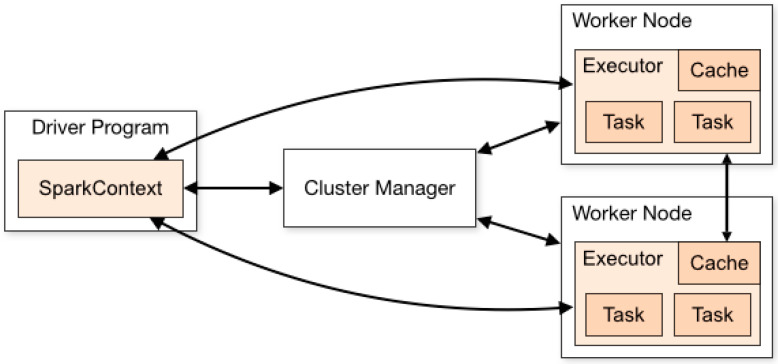
Spark-based cluster operations in the proposed operation.

In the proposed system, we use Apache Spark to add the distributed nature to the proposed architecture. The main function of Spark is to split the dataset into distributed datasets that are resilient to the proposed methodology’s distributed nature. Figure 5 shows the clustering operation in this scenario. The resilient distributed dataset architecture allows us to perform parallel operations on the dataset. The monitor vehicle’s parallel ability to check the co-worker vehicles’ ability speeds up the malicious node detection process. Driver programs are designed and run on monitoring vehicles. These programs help utilize the resources from other vehicles. The head nodes in each cluster are named cluster manager. In the monitoring vehicle’s driver program, an object is named “SparkContext” that helps and connects the manager nodes in each cluster. In this research, we designed a cluster manager node that helps to determine the resource allocation for each node in the cluster programs. So, Spark-based cluster managers are used to determine the resource nodes for each program.

### 3.2. Neural Network in Machine Learning

A neural network is a machine learning-based algorithm designed to mimic the similarities of the human brain. Initially, our goal in this research is to design a classifier. The developed classifier works effectively, as much as the human brain can. We select a single-layer perceptron to design the single-layer neuron operation in the selected research. With the changes in the perceptron, the researchers feel that they have designed many similar neurons in the human brain. In Section 3.3.1, we designed to use a single-layer perceptron, and in Section 3.3.2, we explain the neural network development through this perceptron.

### 3.3. Construction of Network

The VAENT (Visual Analytics for Environmental and Transportation Networks) system creates zones based on clusters. Clusters are groups of similar objects identified using data analysis techniques. By grouping similar objects, zones can be defined to represent specific areas of interest, such as traffic congestion hotspots or pollution hotspots. Using zones helps simplify the analysis of complex data sets, making it easier to identify patterns and trends that may not be immediately apparent. Overall, the construction of zones based on clusters is an important aspect of the VAENT system, helping to facilitate data-driven decision-making in transportation and environmental management. Using unsupervised learning, we construct the zones for the proposed approach. The proposed zone-based effective learning algorithm is constructed. Table 2 selects parameters inside the content placement and selection algorithm.

#### 3.3.1. Perceptron Classifier Based on Single-Layer Operations

**Figure 6:**
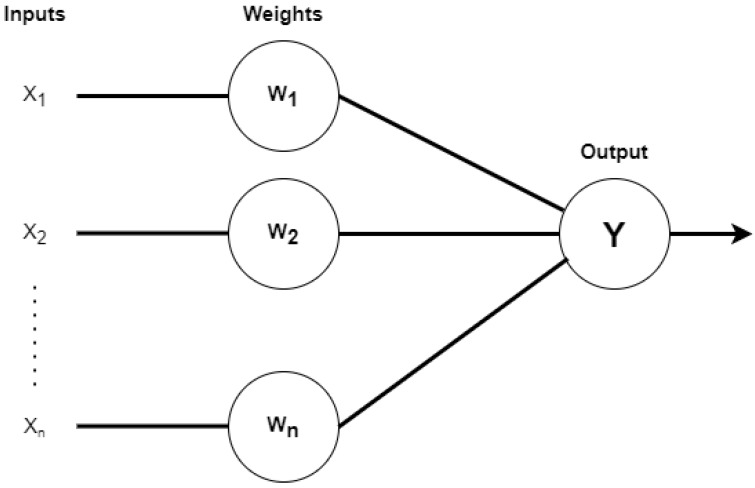
Single-layer perceptron and its operations.

The single-layer perceptron used a binary classification algorithm similar to a single human neuron. The nominated algorithm started with each of the nominated inputs, and weights assigned based on input values. Figure 6 shows the single-layer perceptron and its operations. In this setting the $X_{1},\;X_{2},\;X_{3},\;\ldots \ldots ,\;X_{n}$ are the input values and $W_{1},\;W_{2},\;W_{3},\;\ldots \ldots ,\;W_{n}$ are the associated weights.

The determined values from the layers are determined through Equation (1). These are the multiplicative values determined through the proposed equation.

(1) $$Z=\overset{n}{\underset{i=1}{\sum }}W_{i}\times X_{i}$$

After output in the form of *Z*, the comparison values are conducted against threshold θ. Equation (2) shows the comparison between the threshold and actual predicted values.

(2) $$f\left(x\right)=\left\{\begin{array}{c} 1,\;\;\;\mathrm{if}\;Z>\theta \\ 0,\;\;\;\mathrm{otherwise} \end{array}\right.$$

In step 2, the algorithm defines its predictions based on the dataset provided in this phase of the development steps.

#### 3.3.2. Perceptron Classifier Based on Multilayer Layer Operations

**Figure 7:**
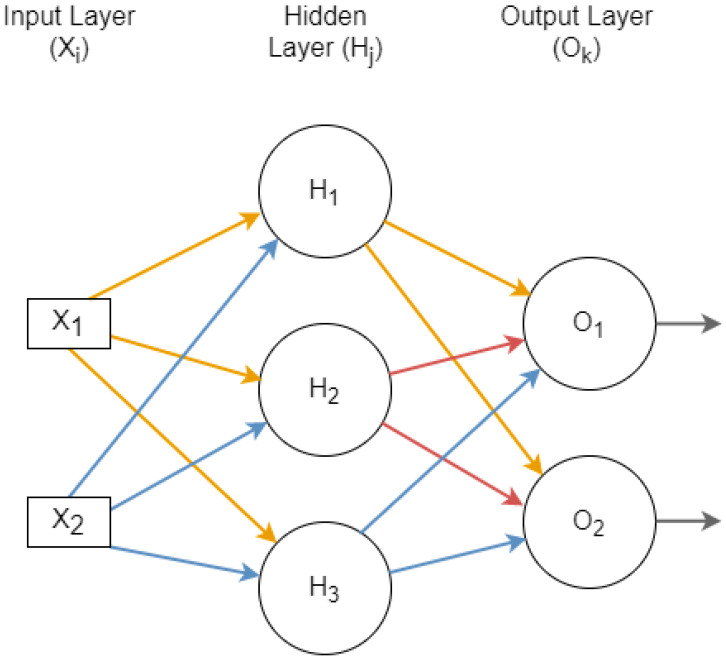
Multi-layer perceptron and its operations.

Researchers group the single-layer perceptron to make a multilayer operational neural network. In multilayer operations, higher predictive outputs can be created to determine the actual positive values from the model. Figure 7 shows the multiple-layer perceptron example. The new model with multiple layers is called the feed-forward neural network (FFNN) or the multilayer perceptron classifier (MLPC). A series of layers are determined through these steps. Each layer is predicted as a single-layer column perceptron. Initially, the input layer is shown in the multiplayer neural network. The input layer is the same size as the dataset’s number of features. The end layer is perceived as the output layer, which shows the required classification results in the given output values. The middle layers are hidden in the multilayer architecture and perform the required classification operations. There is no strict formula to design the required results in the network’s respective input or output operations. The hidden layers are determined through brute force or trial-and-error algorithms.

In mathematics, we perceive that the multilayer architecture is very similar to a single layer with more operations on its hidden layer’s perceptron. The formula in Equation (3) is applied to each layer of the multilayer architecture. The $X_{i}$ is determined by the previous output from the formula. Equation (3) shows the computation of $H_{j}$ through the following formula.

(3) $$H_{j}=f(\overset{n}{\underset{i=1}{\sum }}W_{ij}\times X_{i})$$

The updating is required at weighted values to find $O_{k}$. In the output layer, the $O_{j}$ is used instead of $X_{i}$, to adjust the values from the participated input and output determined. Equation (4) depicts the scenario.

(4) $$O_{j}=f(\overset{n}{\underset{j=1}{\sum }}W_{kj}\times H_{i})$$

### 3.4. Proposed Algorithms

The proposed malicious node detection technique provides the feasibility to control and automate real-time detection. We proposed two algorithms: the first is L-BFGS for the optimization procedure in machine learning, and the second is to find the optimization procedure for the proposed model in VANET machine learning. In the next section, we propose and provide the two main algorithms with their descriptions.

#### 3.4.1. L-BFGS Proposed Segmentation Optimization Procedure

The limited-memory Brayden–Fletcher–Goldfarb–Shannon algorithm (L-BFGS) is selected as the optimization procedure for the proposed model in VANET machine learning. The L-BFGS is a derivation of the Brayden-Fletcher-Goldfarb-Shannon algorithm (BFGS) used for the larger datasets. To explain the proposed method’s main operations, we design two main algorithms, i.e., Algorithms 1 and 2. Algorithm 1 determines the values of $H_{k}\nabla f_{k}$., where $H_{k}$ is the inverse Hessian approximation function value, and the gradient is determined using $f_{k}$ [34]. The main reason to use an approximation Hessian instead of a true Hessian is that it uses L-BFGS as a quasi-Newton method, not a true Newton Method. The values of $H_{k}\nabla f_{k}$ obtained from Algorithm 1 is used to find the directions for $p_{k}$ in Algorithm 2.

**Algorithm** **1:** L-BFGS Recursion to Calculate $H_{k}\nabla f_{k}$
1. **Input:** $q,\;\nabla f_{k},\;H_{k}^{0},\;\rho_{k},\;H_{k},\;\beta ,\;\alpha_{i}\;$2. **Output:** $H_{k}\nabla f_{k}$ values as recursive algorithmic approach**3.** **Steps:**4. Start5. q ← $\nabla f_{k}$6. for i ← k−1, k−2 to k−m Do7.    $\alpha_{i}\leftarrow \;\rho_{i}S_{i}^{T}q$8.    q ←$\;q-a_{i}y_{i}$9. End for10. $r\;\leftarrow H_{k}^{0}q$11. for i ←k−m, k−m+1, ……, k−1 do12.     $\beta \leftarrow \rho_{i}y_{i}^{T}r$13.     r ← $r+s_{i}\left(a_{i}-\beta \right)$14. End for15. $stop\;process\;when\;H_{k}\nabla f_{k}\;\leftarrow r$

**Algorithm 2:** Limited-memory Brayden–Fletcher–Goldfarb–Shannon (L-BFGS)
 1. **Input**: $x_{0},\;H_{k}^{0},\;\mathrm{Memory}\;>0,\;x_{k+1},\;y_{k},f,\;\alpha_{i},\;\left\{s_{k-m},\;y_{k-m}\right\}\;$ 2.  **Output:** Optimization Procedure for the proposed model in VANET Machine Learning **3.**  **Steps:** 4.  Start 5.  start−point ← $x_{0}$ 6.  set−integer:m>0 7.  $Choose:H_{k}^{0}$ 8.  Repeat Steps: 9.        $p_{k}$ ← $-H_{k}\nabla f_{k}$,       ∴from algo-1 10.      $x_{k+1}\leftarrow \;x_{k}+a_{k}p_{k}$   ∴ Wolfe condition satisfaction is $a_{k}$ 11.      if (k>m) then 12.       $discart-vector-pair\;\leftarrow \;\left\{s_{k-m},\;y_{k-m}\right\}\;$ 13.      End if 14.      $compute-vector-pair:\;s_{k}\leftarrow x_{k+1}-y_{k},\;y_{k}\leftarrow \;\nabla f_{k+1}-\nabla f_{k}$ 15.      k ←k+1 16. until−algorithm−converges


In terms of Algorithm 1, $s_{k}$ is described as displacement, and $y_{k}$ is the gradient change. Algorithm 1 contains two loops that are used to update the Hessian matrix. At the start of the initial loop, the current gradient q is determined and steps in length to determine the $\alpha_{i}$. The variable, which is used to determine the values of $\alpha_{i}$, is $\rho_{k}$. The $\rho_{k}$ is computed by using the below-mentioned Equation (5).

(5) $$\rho_{k}=\frac{1}{y_{k}^{T}\times S_{k}}$$

Before the second loop in Algorithm 1, the new value of q is obtained by multiplying the inverse Hessian matrix. Initially, the inverse Hessian is calculated using Equation (6), where I is the initial level Hessian approximation value.

(6) $$H_{k}^{0}=\left(\frac{S_{k}^{T}\times y_{k-1}}{y_{k}^{T}\times y_{k-1}}\right)\times I$$

The final matrices are found by multiplying the $H_{k}^{0}$, and q is referred to as R. The value of R is updated through β values in the algorithm, and the values of $\alpha_{i}$ in Algorithm 1. In the last steps, when the value of R becomes equivalent to $H_{k}\nabla f_{k}$, the algorithm steps and values are determined with the required output.

The time complexity of algorithm 1 is computed by the time required to execute each task of the computational algorithm. The complexity, according to the instruction and number of steps to perform in the algorithms, is O(log n). O(n) is the specified space complexity of the algorithm. The algorithm’s performance denotes it.

#### 3.4.2. Optimization Procedure for the Proposed Model in VANET Machine Learning

**Figure 8:**
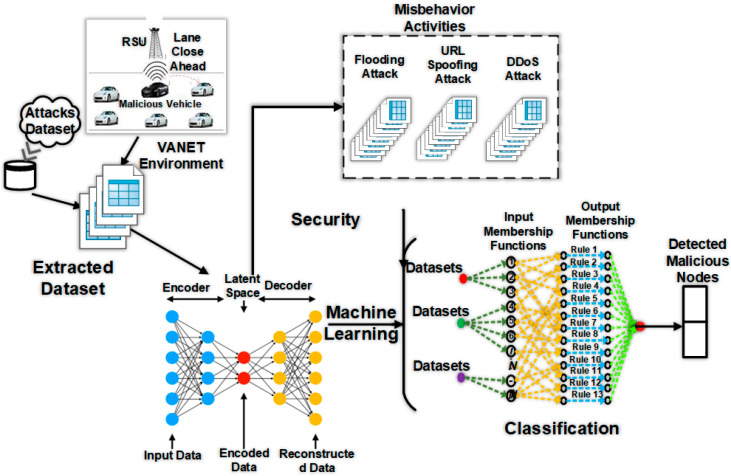
Malicious node detection technique using machine learning.

Algorithm 2 shows the computation of the L-BFGS model. Initially, we set the optimal starting value point, i.e., $x_{0}$, with memory > 0, and initially, the inverse Hessian $H_{k}^{0}$. To find the initial inverse Hessian matrix in Algorithm 2, we apply the same methodology defined in algorithm 1. At this point of the computation, the proposed algorithm computes the direction for the search $p_{k}$, and then updates $x_{k+1}$. After updating $x_{k+1}$, the $\alpha_{i}\;$(step length) must satisfy the Wolfe conditions. By Wolfe condition, we mean that $\alpha_{k}$ is applied for an objective function f. The whole condition under the Wolfe function is shown in Equation (7).

(7) $$f\left(x_{k}+\alpha p_{k}\right)\leq f\left(x_{k}\right)+c_{1}\alpha \nabla f_{k}^{T}p_{k}$$

where *c* is used as a constant among 0 and 1. Algorithm 2 removes the vector pair $\left\{s_{k-m},\;y_{k-m}\right\}$ if and only if *k* is larger than memory m. From Algorithm 1, we mean that $s_{k}$ is the displacement for vehicles, and the change in gradient is stored through $y_{k}$. If these are not verified, then new values related to $s_{k}$ should be computed. The procedure repeats until the algorithm meets at a point of desired optimal value. Figure 8 shows the working of all complete system model.

### 3.5. Logical Workflow of Proposed Methodology

**Figure 9:**
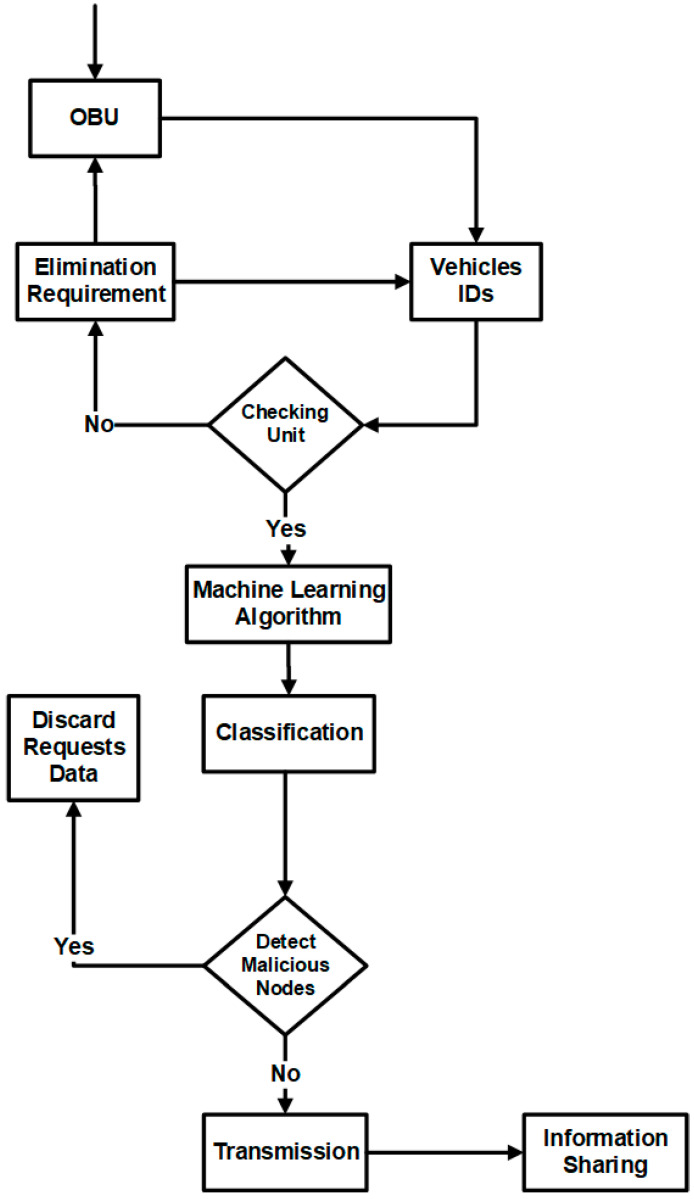
Logical workflow of the proposed methodology.

The proposed technique’s main goal is to detect and classify DDoS-based malicious attacks in V2V and V2I communication in urban environments. Figure 9 elaborates on the workflow proposed methodology. Initially, the OBU received the data requests inside the environment. The vehicle IDs are registered, and elimination requirements are mentioned. After the elimination of the vehicles, the checking units check the units. If no unit is found, the new vehicle ID data is checked. The machine learning algorithms are applied to the dataset to provide the signals. The system uses real-time scenarios to detect malicious nodes using the machine learning approach [50,51]. The classification is applied to the targeted malicious nodes; if the nodes are discarded, the vehicles are distributed over the network. Finally, information is broadcast on the network to identify the DDoS-affected nodes.

### 3.6. Simulation Attack Density Algorithm

Algorithm 3 also defines the attack operations in the simulation setup scenario. The algorithm effectively creates roadside units. IDs are made for implementation and distribution to all nearby vehicles in a simulation environment.
**Algorithm** **3:** Simulations on 10% of attack density for the network environment 1. **Input:** $Messages,\;Wave\;short\;messages\;\left(wsm\right)$ 2. **Output:** Distributed Denial of Service (DDoS) Attack Generation **3.** **Steps:** 4. Start 4. $if\left(simulation-time\;\left(from\;50\;to\;75\right)\right)\&\&\;\left(simulation-time\;\left(from\;210\;to\;224\right)\right)$ then 6.      $for\left(i\;from\;1\;to\;25000\right)\;do$ 7.         sent−Message ←True 8.         wsm←new demo−message(TraCI 9.         $populate\left(wsm\right)$ 10.         $send\left(wsm_{data}\right)\;to\;road_{id}$ 11.         $send\left(wsm\right)$ 12.      End for 13. else  14.      time−last−drove()←simulation−time()

### 3.7. Distributed Multilayer Perceptron Classifier (MLPC) Architecture Development of the Proposed Model

The attack percentages are handled using the architecture development model of the proposed system, called Distributed Multilayer Perceptron Classifier (MLPC). The MLPC uses all the available features from the dataset. According to an example, the simulation environment, which contains 15 vehicles, generates 75 features and is loaded into machine learning models. The initial layer of the MLPC architecture is the feature input. Therefore, the model attempts to identify the DDoS attack occurrence and generates the results in labeled Boolean. The final layer uses two values for the identification of an attack occurrence or nonoccurrence. The five layers of the model outperform the smaller architecture for the proper development of the architecture development. The testing of the dataset is performed using the numbers with a layered architecture. The testing performs better on the third and fourth layers of the NN. Algorithm 4 provides the running of the simulation with the high-performance architecture of the network.
**Algorithm** **4:** Layers determination using Brute Force Method 1.  **Input:** All values from the network  2.  **Output:** Prints F1 score  **3.**
 **Steps:** 4.  Start 5.  for p from 80 to 100 do 6.     for q from 5 to 10 do 7.         for i from 2 to 10 do 8.           layer ←$\;\left[p,\;q,\;k,\;76,\;2\right]$ 9.           $design\_model\left(layer\right)$ 10.           apply (Algorithm 5, $train_{data}$, $test_{data}$) 11.           $fit\_model\left(training\_data\right)$ 12.           $get\_predictions\left(testing\_data\right)$ 13.           $determine\left(\;\right)\;\leftarrow F1\;Score$ 14.           if(f1>0.95) then 15.               $print\left(F1\;Score,\;layers\right)$ 16.         End for 17.     End for 18.  End for

Algorithm 4 collects the results and exports them to a CSV file. In this context, if the combination of layers works multiple times, it runs all the simulation environments. Through this, the developed model has a universal identity and effectiveness rather than operating on only a single attack scenario. Inside the proposed MLPC architecture, the highest values for median and mean over F1 score are [87, 9, 4, N, 2], where N is the number of features given through the dataset. Algorithm 4 it refers to the input layer, and 2 refers to the output layer of the model. From the dataset, we use all the feature-labeled columns as labels. The values 87, 9, and 4 show NN’s hidden layers. In addition, 91.5% is the average F1 score, and 95.9% shows the median score over the network architecture. Algorithm 5 decides the training and testing dataset, splitting and fine-tuning the dataset according to the reference model. The training and testing dataset is then provided to Algorithm 4 for ML algorithm classification.
**Algorithm 5:** Decision about training and testing dataset 1.  **Input:** Complete Dataset  2.  **Output:** $traing_{data},\;test_{data}$ **3.**
 **Steps:** 4.  Start 5.  $Split(traing_{data},\;test_{data}$). 6.  $train^{data}=Rand\_method\left(data^{complete},\;train\right)$ 7.  $test^{data}=Rand\_method\left(data^{complete},\;test\right)$ 8.  $Model{\left(\right)}_{train}^{data}$ 9.  $model\_train\left(data^{test}\right)$ 10.  Repeat steps 4, and 5 11.  $Accruacy_{improve}=param()$ 12.  If(accuracy=satisfied()) 13.    $data^{predict}=new_{data}$ 14.  End

## 4. Results

In this chapter, we discuss the results to achieve the main objectives of the research from the proposed model. We have considered security as the key element that we achieve through the proposed model, with adequate consideration of the provided framework model. Three key elements are needed to achieve the proposed zone-based content caching approach in VANET for congestion control using machine learning. The key parameters are cache hit ratio, throughput, prediction accuracy, and average delay.

### 4.1. OMNeT++ Indication

To evaluate the performance of vehicular communication and security management, OMNeT++, NSL-KDD, NS-2/3, and UNSW simulators were discussed in Section 2 for introduction purposes [52]. In addition to all of these tools, OMNeT++ is one of the backbone simulators for vehicular communications. To handle advanced-level vehicular communication systems, OMNeT++ is introduced. In the simulation environment, we introduce mobile devices, SUMO, and Veins to be experts in handling such simulation experiments. In OMNeT++, SUMO is considered to control the traffic simulations to generate the normal vehicles and mobility inside the vehicles. As SUMO and OMNeT++ work together to generate full traffic control, Veins is used as the glue to connect both and make a complete vehicular communication system for the proposed methodology. In this thesis, the OMNeT++ is designed to enhance 1-Hop broadcasting among roadside units (RSU) and vehicles [53]. This is performed to distribute the messages to each node in the range of the sender vehicle.

### 4.2. Background Information and Simulation Settings

The Ubuntu virtual machine designs and develops all vehicular virtual network simulators. During installation, the Veins are designed with built-in maps and a simulation system for vehicular communication. In this simulation, we use 195 vehicles, and all drive in the same direction. In this simulation, 73 s are used when the incident occurs. The total duration is 50 s. The incident causes the other vehicles to alert and react with other vehicles, causing a traffic jam. A total of 200 s were lost during this simulation. A single RSU is also involved in this step for processing. This simulation is used as starting point for such simulations. To simulate DDoS attacks, the distance between the nodes is variable due to the movement of vehicles on the highway. The maximum number of 200 nodes (vehicles) are used in the simulation environment. The connectivity inside the network is a 5G wireless communication architecture. The attack vehicles are used to perform the attack simulation environment scenarios.

**Figure 10:**
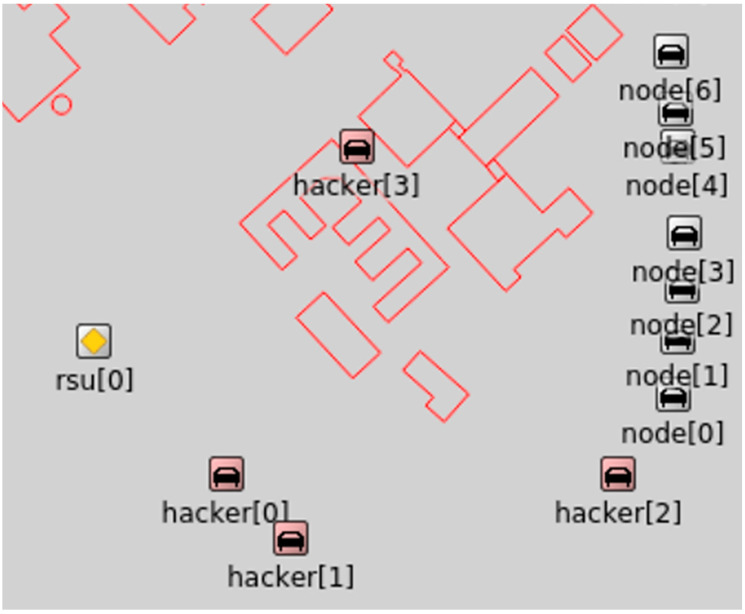
Veins/OMNET++ attacker simulation environment.

In the default simulation setup, a sequence of revisions was adjusted to make it feasible for this research. The total simulation time was increased to 380 s. According to the simulation of vehicles, 15, 20, 25, 30, and 35 were made in the setup. In the previous setup, four parked vehicles were used as an attack, using DDoS on RSU and other vehicles. Therefore, these vehicles are utilized in an attack scenario. These were considered otherwise the same as in the attack scenario for possible consideration. The vehicles utilized in the attacks were considered the same as the possible mobile vehicles. Attackers use these vehicles as zombies, which means that other than attacking, these vehicles normally communicate in the network. At 73 s of the simulation program, the accident was considered, and no change was made to this arrangement. Figure 10 shows the OMNeT++ simulation setup for the proposed methodology. In the simulation environment at 11, the attackers were marked as “hackers,” and normal communication vehicles were marked as “nodes”. Equation (7) computes the accuracy values.

(8) $$\mathrm{Accuracy}\;=\frac{\mathrm{Number}\;\mathrm{of}\;\mathrm{Classified}\;\mathrm{Samples}}{\mathrm{Total}\;\mathrm{Samples}}\;$$

We developed seven versions for each attack in a simulation environment to check the proposed system’s attack handling density. Initially, we set up 10% for the first simulation setup. This means a 10% simulation time was checked to set up the attack simulation time. After that, we increased the attack density by 10% for every simulation to test the proposed system’s performance. Table 3 shows the simulation time for the attack, the first attack duration, and the second attack duration in percentage.

In the simulation setup, those attacks which run only on a 10% density of attack were run from 50 to 74 s and 210 to 224 s. At 50 s, the first attack started on every attack density. At 210 s, the second attack started for every density. All stacks were started, but the attack started at 174 s is at 70% of the attack density. In this stipulated simulation time, we sent 25,000 wave short messages to every vehicle in the simulation time. The wave short messages code used to send short messages is in traCIDemo11p.cc in the Veins setup. In the simulation, the malicious changes were discussed in the handlePositionUpdate method for effective simulation setup. Algorithm 3 defines the simulation setup and other main methods in this setup.

### 4.3. Dataset Preparation/Generations

The dataset is selected as a DDoS attack scenario from the OMNeT++ interactions and its simulation environment. Generating datasets for malicious nodes in VANET is an important aspect of studying the security of vehicular communication networks. Using SUMO++ and Veins simulators, it is possible to simulate and analyze the behavior of malicious nodes in the network. These simulators enable researchers to generate realistic scenarios where malicious nodes may compromise the integrity and confidentiality of the data transmitted in the network. Such datasets can help improve the security of VANET by identifying vulnerabilities and devising effective countermeasures against potential attacks.

The extracted and cleaned CSV dataset file is handled using Jupyter notebook IDE through the Spark read method. The columns inside the dataset are shifted toward the real values before being loaded into the PySpark.ml machine learning models. The attack on the dataset column is shifted towards the handling of the model of labeled data using PySpark. The feature vector method is adopted to split the dataset into training and testing datasets. In our case, we split the dataset into 50% for training and 50% for testing on the available dataset. The dataset is prepared to provide the vector representation of data-like libraries for the PySpark library to develop the ML-based models. The dataset is loaded into machine learning models and transformed into the required columns of the dataset, and then the model is used as input for training and testing.

**Figure 11:**
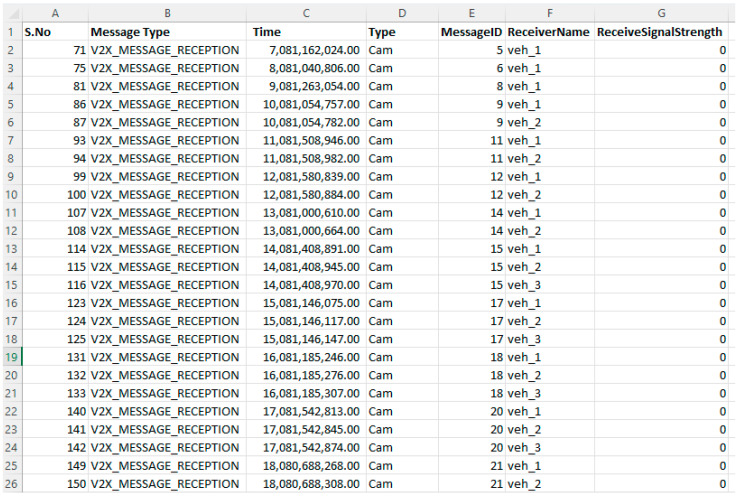
Message reception dataset sample.

**Figure 12:**
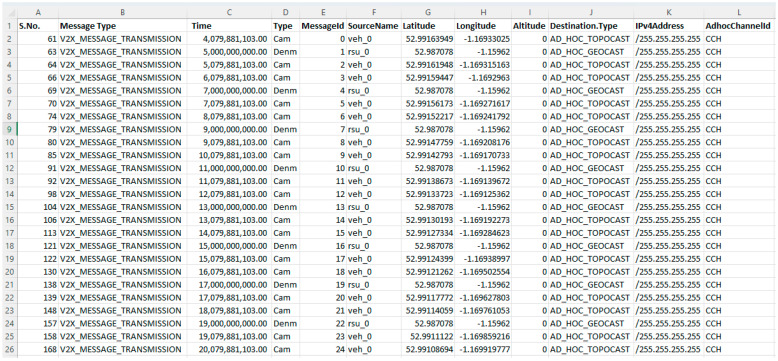
Message transformation.

**Figure 13:**
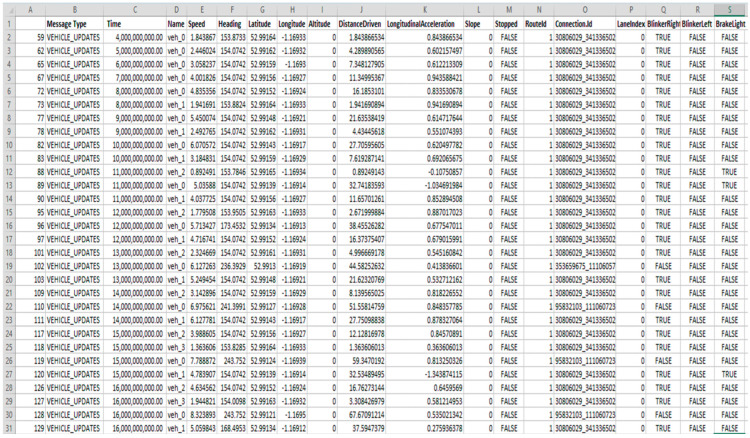
Samples from the vehicle updates dataset.

The work of machine learning algorithms is dependent on datasets through which the algorithms train and test. The features inside the dataset allow the machine learning models to work and detect malicious nodes effectively. The dataset used in this work was gathered from real-time scenarios under OMNET++ and SUMO with some online repositories, including Kaggle and UCI. Some datasets were downloaded from the IEEE dataset repository and named the malicious nodes dataset. The characteristics of the dataset include message ID, message type, time, type, message ID, receiver name, receiver signal strength, no received requests, number of generated requests, source name, longitude, latitude, destination, IPv4 addresses, channel, slope, stopped, route ID, connection ID, lane index, blink left, blink right, break left, break right, and number of decisions. The main dataset were grouped into i-e, message reception, message transformation, and vehicle updates. We use 20,030 lines of the dataset, 10,030 samples for training the models, and 10,000 samples for testing purposes. The size of the dataset affects the accuracy of the ML model. Figure 11, Figure 12 and Figure 13 show the dataset values.

### 4.4. Simulation Results

#### 4.4.1. Comparison of Models for Attack Densities

The Jupyter Notebook on the HP Notebook compares the architecture’s performance with existing architectures using common ML models. The machine is equipped with Intel Core i7 10th G Processors with 16 GB of RAM. The model was compared using random forest, logistic regression, support vector machine, and gradient boosted tree. In this context, we adopted the training data with 70% and the testing data with 30%, splitting from the main dataset. In addition to the random forest ML model, the other models train over 100 training iterations. Inside the PySpark ML, random forest does not provide an option to restrict to 100 training iterations. Three tree depth is set for random forest as a maximum depth. The PySpark ML module is used to build all the models over the network. PySpark is the main ML library developed by Apache Spark over the other machine learning modules. The library can use the apart cluster values over the network. The main parameters are considered to evaluate the performance, such as F1 score, precision, accuracy, and recall, for comparing the performance of these models.

The sum of true positive (TP) and true negative (TN) values is used to find the ML model’s accuracy. All possible outcomes are divided into these values of TP and TN. False positive (FP) and false negative (FN) are also used to evaluate the performance of these network resources. Equation (8) shows the accuracy computation of ML models.

(9) $$Accuracy=\frac{TP+TN}{\left(TP+TN\right)+\left(FP+FN\right)}$$

To compute the precision values, the TP is divided by the sum of FP and TP in Equation (9).

(10) $$Precision=\frac{TP}{FP+TP}\;$$

Equation (10) shows the computations of the recall values for the model. It shows that TP is divided into FN and TP.

(11) $$Recall=\frac{TP}{FN+TP}\;$$

According to Equations (9) and (10), the precision and recall are computed. The F1 score shows the weighted average of both values. Equation (11) shows the computation of these values to divide the multiplication of results from Equations (9) and (10) over the sum of results from Equations (9) and (10). Then computations from these values are multiplied by two for the F1 score. Equation (11) shows the F1 score computation.

(12) $$F1\;Score=\frac{Precision*Recall}{Precision+Recall}\;$$

#### 4.4.2. Model Accuracy Results

**Figure 14:**
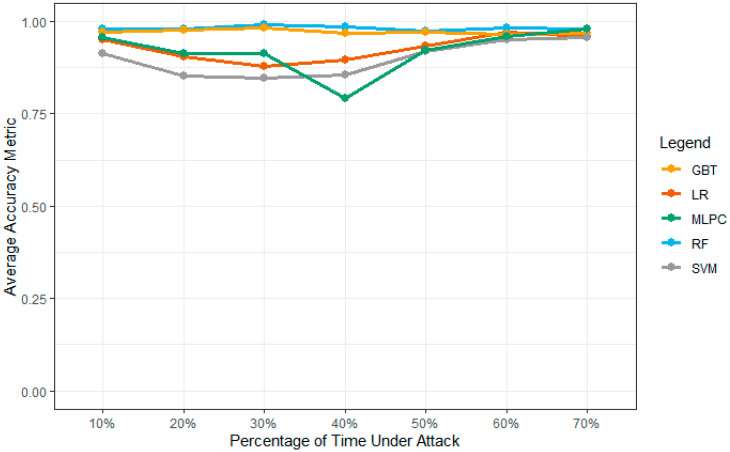
Models’ accuracy over multiple nodes.

**Figure 15:**
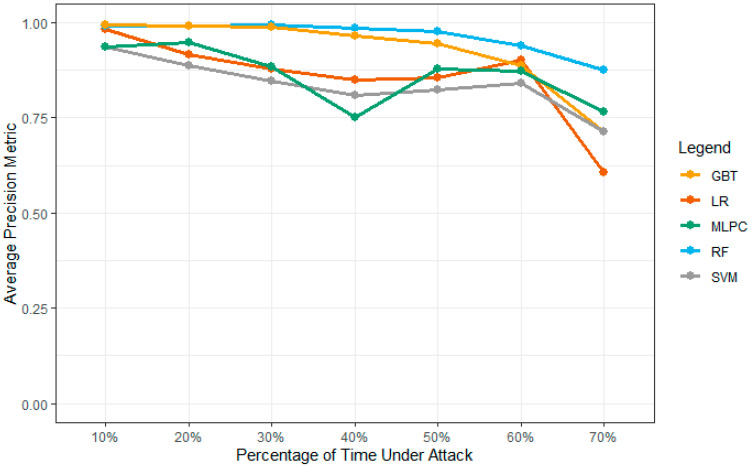
The precision of the models for the proposed system.

Consistency is one of the main reasons to implement the values of these functions using the PySpark ML library. The implementation provides the correct computations of these values using Python code. Table 4 shows the values of the model accuracy results under different machine learning models. These simulations are computed and collected over the five-simulation environment to collect these vehicles over the network environment. Figure 14 shows the accuracy value of the model over the multiple attack densities. The weak point of the MLPC model can be observed using the below-mentioned graph. Overall, the attack densities in the production model show an F1 score. The overall model is not as effective as the densities, as shown in their results. According to the analysis in Figure 15, GBT and RF show higher accuracy values for all network densities. The proposed system outperforms the SVM and LR models with a 40% attack density over the network performance layers.

#### 4.4.3. Model Precision Results

The precision for each model should follow the trends to find the accuracy values over similar trends. RF and GBT outperform other models with 60 to 70 percent attack densities over GBT. The proposed MLPC performs better in training the LR models. The comparison is performed over the number of vehicles within the network which accounts for over 40% of the scores. Based on the regression values, the SVM does not perform well compared to other models for the final and effective confirmation of the values from these models. Figure 15 shows the precision values over all attack densities for the final and effective model comparisons. Table 5 shows the precision values from GBT, LR, MLPC, RF, and SVM models.

#### 4.4.4. Model Recall Results

**Figure 16:**
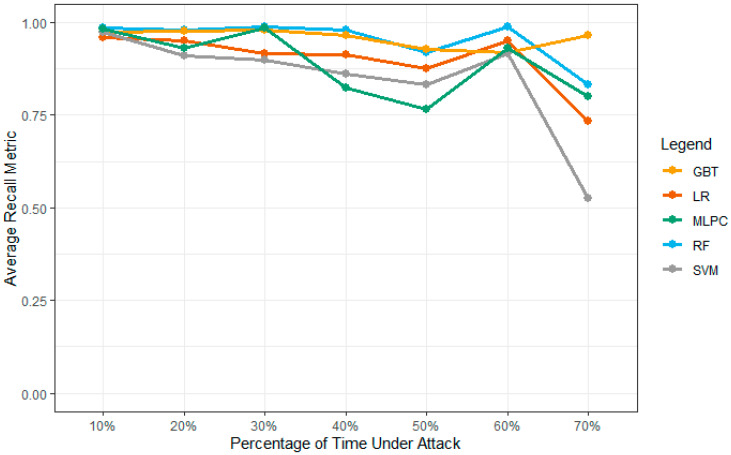
Comparison of model recall values over the network performance.

Table 6 shows the values of the model average recall metric results under different machine learning models. These simulations are computed and collected over the five-simulation environment to collect these vehicles over the network environment. The model recall is considered for all ML models except MLPC in Figure 16. The models such as GBT, RF, and MLPC, performed best over the network attack percentage and average recall metric at a 40% attack density of MLPC. At 60%, all the models show the highest recall results over density values, and then over 50% of these results show the highest recall values for all models. The GBT drops over 70% of the network attack density for network performance management. Based on the results, it clearly shows that LR performs better as compared to other results and outperforms over the same network density values. The SVM produces similar results to LR, but the results are comparatively weak compared to SVM. Figure 16 shows the complete results of these network values.

#### 4.4.5. F-1 Score Results for Models

**Figure 17:**
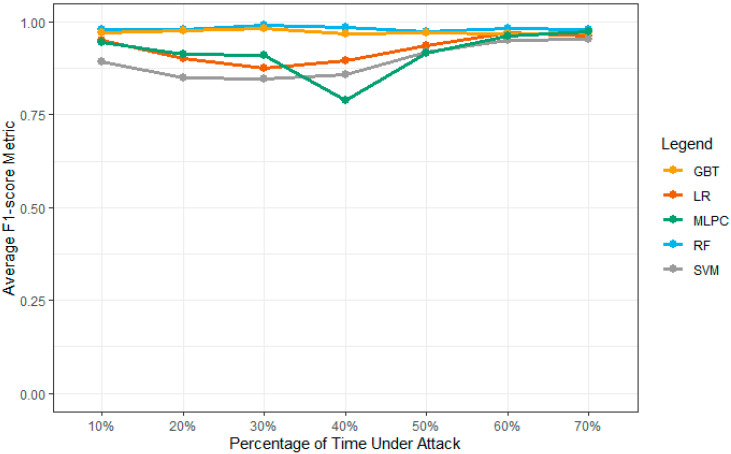
F1 Score values for multiple models for network attacks.

Table 7 shows the values of the model average F-1 score results under different machine learning models. These simulations are computed and collected over the five-simulation environment to collect these vehicles over the network environment. The F-1 score depicts the performance of the model accuracy measurement over the dataset. Figure 17 shows the F-1 score values for all ML models over the dataset for network attack density and attack computations. The GBT and RF show the highest level of the F-1 score across all levels of simulation parameters. According to the results, the MLPC shows lower or equivalent metrics compared to GBT and RF. On the 40% attack density values, the MLPC shows lower results compared to other results in the simulation environments. The SVM and LR show comparatively good results over the 40% attack density values for the proposed and network models. At the 70% attack density, the models outperform with better results compared to their current score and results.

### 4.5. Cluster-Based Training and Testing Time for the Proposed Network Architecture

**Figure 18:**
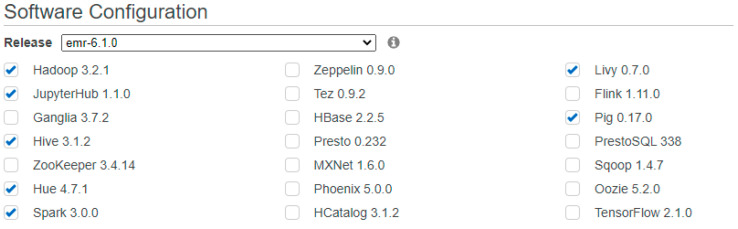
Amazon MapReduce configuration information.

We have applied PySpark, which is based on distributed technology. Figure 18 shows the complete configuration. The values gained from the vehicles show the computations from these vehicles. In addition, 50% of the attacks are stored online using AWS services to simulate the whole process. The Amazon MapReduce tool sets up the servers for PySpark cluster running and schemes storage. AWS supports the Amazon MapReduce cluster to run the Jupyter Notebook. The single controller node and other computational nodes are considered in the cluster transformation. The server is configured with 4 Core and 16 GB of memory to deliver the actual values generated through the produced results.

### 4.6. Computational Speed and Node Amount of MPLC

**Figure 19:**
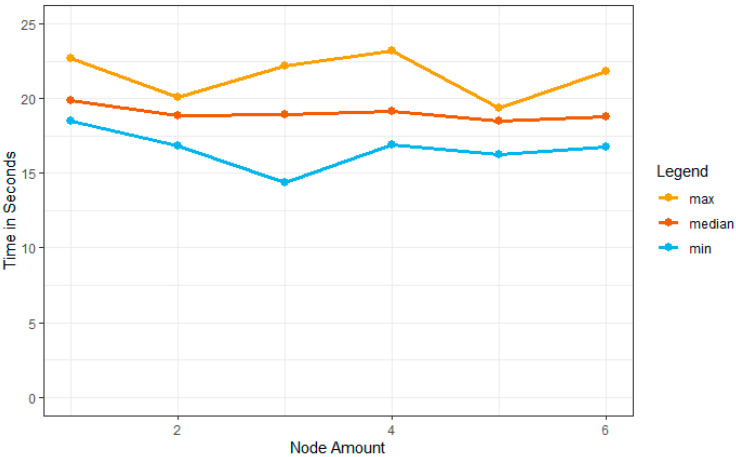
Vehicle node amount and computational speed in different scenarios.

The training and classification/prediction instance configuration determine the computational performance. The six different AWS servers are used to adapt the total performance of these nodes. One to six total controller nodes are considered in the simulation setup. In this simulation, 35 vehicles are used to analyze the network’s performance. Ten different times are noted with the simulation environment’s ability to configure the instances. Figure 19 shows the algorithm utilization and training running time for every node. The additional nodes we try to add impact the information provided and the running time performance details. Their median time had decreased when nodes were included in the simulation setup.

### 4.7. Comparison of Results with Existing Techniques

**Figure 20:**
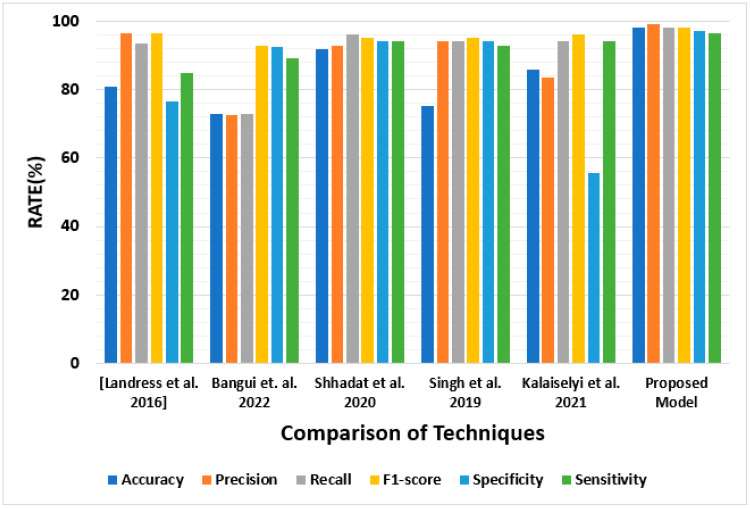
Comparison of the proposed model (IVSDDM) with previous research works [41,42,43,44].

The results are compared with existing methodologies. This shows that the proposed approach performs better than the previous approaches mentioned in the results. The evaluation parameters are accuracy, precision, recall, F-1 score, specificity, and sensitivity. Table 8 shows the results compared to the proposed approach. Alongside Table 8, Figure 20 shows the complete comparison of the presented results with similar findings from the literature discussed and enlisted in the present scenario. The work presented in Table 8 and Figure 20 shows that our proposed approach works better compared to other techniques presented in [40,41,42,43,54].

## 5. Conclusions and Future Research

### 5.1. Conclusions

VANET is one of the most demanding network architectures that provides high-level data-sharing connections between vehicles. In this methodology, we simulate real-time malicious node detection using machine learning. In this research, we focused on the VANET architecture with the ability to contribute and provide reliable services for high-level real-time malicious node detection. We call this misbehavior detection using machine learning techniques. We set up the environment with an attack scenario over a density of 10% to 70% from the external environment. We adopted the GBT, LR, MLPC, RF, and SVM to compare the results of machine learning models. The results show that the proposed approach presents accuracy, precision, recall, F1 score, specificity, and sensitivity with 98%, 99%, 98%, 98%, 97%, and 96.6% from the proposed results. RF is recommended for better misbehavior detection results. The results accurately predict that the proposed architecture effectively handles the research in the VANET environment.

### 5.2. Future Research

In the future, we plan to work on further analysis by adopting deep learning-based advanced persistent threats (APT) to detect and protect the network. The future recommendations also adopt more accurate results prediction analysis and provide a brief justification for the results and outcomes. Other possible future research directions on real-time malicious node detection using machine learning are:

- **Developing novel machine learning algorithms for malicious node detection:** Researchers can develop novel machine learning algorithms specifically designed for VANETs. These algorithms can be optimized for real-time processing and high accuracy.
- **Improving feature selection:** Feature selection is a critical step in the machine learning process. Researchers can investigate which features are most relevant for detecting malicious nodes in VANETs. They can also explore new features that have not been previously used for this purpose.
- **Analyzing the impact of different attacks on malicious node detection:** Various attacks can be launched on VANETs, such as jamming, impersonation, and denial-of-service attacks. Researchers can analyze the impact of different attacks on the performance of machine learning algorithms for malicious node detection.
- **Designing a hybrid approach:** A hybrid approach that combines multiple machine learning algorithms can be developed to improve the accuracy of malicious node detection. For example, an ensemble of algorithms such as decision trees, neural networks, and support vector machines can be used to detect malicious nodes.
- **Investigating the trade-off between detection accuracy and computational complexity:** Real-time processing is crucial for malicious node detection in VANETs. However, processing large amounts of data in real time can be computationally complex. Researchers can investigate the trade-off between detection accuracy and computational complexity to develop a system with high accuracy and low computational overhead.
- **Evaluating the system’s robustness to different network scenarios:** VANETs are subject to different network scenarios, such as varying traffic densities, topologies, and mobility patterns. Researchers can evaluate the robustness of the proposed system in these different scenarios to ensure that it works effectively in different environments.
- **Testing the proposed system in a real-world environment:** The proposed system should be tested in a real-world environment to evaluate its effectiveness in detecting malicious nodes in VANETs. This can involve setting up a testbed and collecting data from real vehicles to evaluate the system’s real-time accuracy and efficiency.

## Figures and Tables

**Table 1:** Comparison of different existing state-of-the-art techniques under multiple parameters.

S. No.	Author and Year	Algorithm Used	Real-Time	Dataset	Environment Setup	Tool Used	Malicious Attack	Accuracy
1	Eziama et al. [37]	ANN	No	NG-SIM	VANET	Matlab	Misbehavior	97%
2	Bangui et al. [41]	Deep belief network technique	No	Online	VANET	NS-2	Trust Enhancement	96%
3	Kalaiselvi et al. [43]	SVM	Yes	KDD 1999	VANET	Matlab	DDoS	85%
4	Kaur et al. [44]	SVM	Yes	Self-generated	VANET	SloMoSIM	Misbehavior	92%
5	Patil et al. [45]	IBK, RF, BN, and Boost-1	No	Self-generated	VANET	Weka	Misbehavior	93%
6	Haung et al. [46]	SVM	No	Self-generated	SDVN	Flood-Light	DDoS	97%
7	Karthiga et al. [47]	K-Mean	Yes	Self-generated	VANET	NS-3	DDoS	95%
8	Nayak et al. [48]	Graph-based machine learning technique	Yes	DFRA	VANET	SUMO	Malicious Node	95%
9	Sharma et al. [49]	Cluster algorithm and fest classification	No	Online Kaggle	VANET	ML	Malicious Node	96.5%
10	Proposed techniques	GBT, LR, MLPC, RF and SVM	Yes	Self-generated + Kaggle	VANET	ML and NS-3	Misbehavior + DDoS	99%

**Table 2:** Parameters with the descriptions used for the proposed methodology.

S.No.	Parameters	Definition	Description
1	$Z_{i}$	Zone declared in VANET, i.e., Z1 for zone 1, Z2 for zone 2, and so on.	In every cluster, the RSU holds the data and obtains the system feedback to be developed to capture the network. The clusters are according to the RSU numbers.
2	$CM_{y}^{D}$	Central manager for vehicle data storage.	The master central BS has complete data and architecture implementation of the real-time machine learning applications. It provides the safety and security requirements along with the methodology to implement the results.
3	$CM_{y}^{dec}$	The BS starts the communication and monitoring.	The backend BS is responsible for deciding and architecting the communication architecture collaboratively and effectively. The central manager decides the collaboration and centralization architecture to be affected.
4	RSU	RSU is held responsible for making clusters of ideas and making the network.	The responsibility of the RSU is to make it more vulnerable to attacks and make it more reasonable for deploying the respected scenario.
5	CT	BS is used to obtain central storage responsibilities.	The complete content of the network is only accessible to the network architecture, which has the ability to demonstrate knowledge and experience perspectives. It enhances and controls the communication architecture under such a scenario.
6	$C_{zi}\;$	Congestion of zone	The congestion in every zone is balanced through the proposed techniques. The congestion is related to the content and its requests for content placement and enhancements.
7	CH	Cluster heads for zones	Every zone holds a selected cluster head, which is part of the cluster, and makes effective cluster collaborations to discuss and provide the cluster-based implementation policies. The discussed results provide effective zone-based content pre-caching and enhancement strategies.
8	$X_{red}\;$	Zone radius	Every zone holds a radius that consumes the zone area and provides the system enhancement features to collect and discuss the resulting privileges.
9	ML	Machine learning algorithms for vehicles.	We adopted a machine learning vehicles-based approach to handle the content congestion and enhanced the features for the collaborative workings.
10	Data Exchange	The data is the exchange between the network and different parties for network performance.	The content is the exchange between RSU, BS, and vehicles before and after the attacks. These devices provide the full flexibility to exchange data with high-volume perspectives.
11	$V2V_{com}$	Communication point from vehicle to vehicle.	The communication point from the vehicle addresses provides the collective methodology for the results and discussions to elaborate and provide the working methodologies effectively.
12	$V2I_{com}\;$	The communication with vehicular output architecture.	The complete network permission and vehicle communication architecture is provided with the ability to demonstrate and enhance the total communication between the vehicle to infrastructure communication.
13	$R_{r}$	Requested content of size r	The content requested by the consumer node is size r, where r is the threshold value set in the algorithm to provide effective caching placement and implementation strategies.
14	$D_{r}$	Delivered content of size r	The content, which is delivered to the requested vehicles, is size r.
15	$Req_{data}$	Requested content with data requested.	This shows the request for the data and content from the requested node toward the content placement and enhancement strategies.
16	$DM_{Node}^{Z}$	Data rate among manager nodes and vehicles.	The data rate for the manager node and vehicles shows at which rate the data is delivered among these nodes for proper implementation.
17	$Z_{table}$	Intra cluster entries table	The table stores all the communication entries found between the communication of RSUs and vehicles between different clusters.

**Table 3:** Attacker simulation time in setup.

Attack Percentage	Duration (1 Attack) Sec	Duration (2nd Attack) Sec
10	50 to 74	210 to 224
20	50 to 74	210 to 264
30	50 to 112	210 to 264
40	50 to 150	210 to 264
50	50 to 150	210 to 300
60	50 to 150	210 to 237
70	50 to 150	174 to 340

**Table 4:** Model Accuracy result of the networks comparison.

Time Percentage under Attack	ML Algorithms				
	GBT	LR	MLPC	RF	SVM
10%	0.92	0.89	0.87	0.94	0.84
20%	0.94	0.83	0.85	0.95	0.79
30%	0.95	0.85	0.86	0.98	0.82
40%	0.94	0.92	0.78	0.97	0.88
50%	0.95	0.94	0.87	0.96	0.89
60%	0.95	0.92	0.91	0.97	0.92
70%	0.94	0.93	0.97	0.97	0.93

**Table 5:** Model Precision Results of the network and comparison with other networks.

Average Precision Metric	ML Algorithms				
	GBT	LR	MLPC	RF	SVM
10%	0.986	0.975	0.864	0.989	0.864
20%	0.987	0.897	0.874	0.978	0.875
30%	0.987	0.864	0.861	0.990	0.869
40%	0.982	0.787	0.750	0.979	0.921
50%	0.942	0.821	0.874	0.974	0.924
60%	0.897	0.883	0.875	0.941	0.799
70%	0.742	0.764	0.765	0.875	0.751

**Table 6:** Model recall results under different networks.

Average Recall Metric	ML Algorithms				
	GBT	LR	MLPC	RF	SVM
10%	0.96	0.95	0.97	0.98	0.96
20%	0.97	0.955	0.92	0.98	0.87
30%	0.98	0.93	0.98	0.99	0.88
40%	0.97	0.94	0.83	0.98	0.93
50%	0.92	0.93	0.78	0.95	0.89
60%	0.91	0.96	0.89	0.99	0.92
70%	0.96	0.74	0.82	0.87	0.67

**Table 7:** Model F-1 results under different networks.

Average F-1 Score Metric	ML Algorithms				
	GBT	LR	MLPC	RF	SVM
10%	0.96	0.93	0.92	0.97	0.88
20%	0.97	0.88	0.89	0.98	0.87
30%	0.98	0.86	0.89	0.99	0.89
40%	0.95	0.89	0.78	0.99	0.88
50%	0.94	0.93	0.91	0.97	0.93
60%	0.96	0.95	0.94	0.99	0.92
70%	0.97	0.95	0.96	0.98	0.94

**Table 8:** Comparison of the results with existing approaches.

Reference	Parameters					
	Accuracy	Precision	Recall	F1 score	Specificity	Sensitivity
[40]	80.8	96.5	93.5	96.4	76.7	84.9
[41]	73	72.7	73	93	92.4	89.3
[42]	92	93	96	95	94.3	94
[54]	75.3	94	94	95	94	93
[43]	85.9	83.7	94	96	55.6	94
Proposed Model	98	99	98	98	97	96.6

## Data Availability

Not applicable.
